# A Holistic, Data-Driven Approach to Diversifying Alpha Omega Alpha (AΩA) Honor Society Electees

**DOI:** 10.7759/cureus.102163

**Published:** 2026-01-23

**Authors:** Seema Baranwal, Carolyn Giordano, Leon McCrea, Jennifer Hamilton, Kathleen Ryan, Bisan Salhi, Roshell Muir, Amy Fuchs

**Affiliations:** 1 Medicine, Drexel University College of Medicine, Philadelphia, USA; 2 Family, Community, and Preventive Medicine, Drexel University College of Medicine, Philadelphia, USA; 3 Emergency Medicine, Drexel University College of Medicine, Philadelphia, USA; 4 Faculty Affairs, Wake Forest University School of Medicine, Winston-Salem, USA

**Keywords:** academic recognition, holistic review, honor societies and awards, honor society selection process, medical education, medical school honors

## Abstract

Recently, some medical schools eliminated their Alpha Omega Alpha (AΩA) chapters because of concerns that AΩA academic criteria led to bias in student selection. In 2020, the AΩA board changed the constitution to permit individual member schools to determine how their electees would be chosen. Thus, the relative weight of AΩA’s selection criteria could be defined by the individual schools, allowing for a more holistic approach to selection. This study at a single large private medical school examines student AΩA electee rates before and after the implementation of holistic review using an institutional platform, MyPortfolio (MP; Drexel University College of Medicine, Philadelphia, USA), to holistically select electees. A cross-sectional study design was used to assess demographic data of medical students considered for AΩA membership election between 2016 and 2025 and compared pre-MP (2016-2020, N = 220) with post-MP (2021-2025, N = 270) electees. Chi-square analyses demonstrated statistically significant increases in the representation of racially and ethnically underrepresented in medicine (URiM) students (2.73% to 10.05%, p < .01), first-generation students (0% to 6.27%, χ²(1, N = 491) = 12.48, p < .001), and students from lower socioeconomic backgrounds (12.73% to 21.77%, χ²(1, N = 491) = 6.21, p < .001). The study found that a standardized platform, such as MP, can function to perform a holistic review of AΩA electees in our college. These findings highlight that a more equitable AΩA election may be achieved using a holistic review and improving professional opportunities for medical students of all backgrounds.

## Introduction

Membership in the Alpha Omega Alpha (AΩA) Honor Society is a longstanding hallmark of academic achievement of medical students [1]. Although AΩA selection has evolved to include qualities such as leadership and service to the school and community, studies have shown racial, ethnic, and socioeconomic disparities in AΩA membership [2,3]. The literature also highlights how these disparities negatively affect underrepresented students who may aspire to careers in academic medicine [2], as limited access to this prestigious membership could hinder their pursuit of advanced educational and professional opportunities.

In 2020, AΩA changed their charter to permit individual member schools to “identify students who, based on merit, demonstrate the characteristics of becoming excellent physicians and are aligned with AΩA’s mission and values and the school’s determination” [4]. This change allowed for a holistic review of students’ achievements.

Considering the AΩA charter revision, we began utilizing MyPortfolio (MP; Drexel University College of Medicine, Philadelphia, USA), a centralized institutionally developed platform, to measure and prioritize academic, research, service, and leadership achievements. Unlike the previous selection method that relied heavily on academic metrics, MP facilitates a more holistic evaluation of student achievement. This study hypothesized that the utilization of MP to holistically evaluate medical students would yield a difference in the demographic representation of AΩA electees at our medical school.

Research question

The research question we sought to answer was: Does a holistic review of medical school Alpha Omega Alpha (AΩA) honor society candidates better reflect the broader demographics of the class compared to traditional academic metrics?

## Materials and methods

MyPortfolio (MP)

MyPortfolio (MP) was developed in 2013 to centralize the tracking of medical students’ extracurricular involvement and to improve the accuracy and efficiency of preparation of the Medical Student Performance Evaluation (MSPE). Our students are required to enter their data into MP annually, along with relevant descriptors (e.g., type of activity, leadership involvement, time commitment).

Since its inception, MP has gone through iterative improvements to ensure ease of engagement and accuracy of data collection. Beginning with the class of 2021, MP has served as the primary mechanism to collect and utilize data for the AΩA selection process.

Participants and demographics

Our institution is a very large private allopathic medical school in the United States (US) with over 1,200 medical students enrolled. Our student body is diverse in background and professional interests, a critical strength of our institution and a pillar of our educational mission. Nevertheless, tracking our students’ achievements and rewarding them accordingly is challenging and a primary driver of the comprehensive MP processes.

Study design

In this cross-sectional study, we used the admissions database to obtain de-identified demographic data of students considered for AΩA election between 2016 and 2024. We compared AΩA election in the pre-MP (classes of 2016-2020) and post-MP (classes of 2021-2025) periods based on the Association of American Medical Colleges (AAMC) self-reported characteristics, gender, race, ethnicity, first-generation, and socioeconomic status (SES), with SES 1 and 2 designated as having lower socioeconomic status [5]. We employed the Strengthening the Reporting of Observational Studies in Epidemiology (STROBE) reporting guidelines [6]. The Institutional Review Board of Drexel University approved this study.

AΩA candidate selection

Students are elected to AΩA by a committee comprised of faculty members of AΩA. In the pre-MP period, students were elected based on a weighted average of grades (20% year 1, 20% year 2, and 60% year 3 grades). The committee was blinded to student names.

In the post-MP period, the committee selected four overarching selection criteria that would be utilized to guide our Chapter’s AΩA selection, each of which could be accurately quantified through curricular assessment or the MyPortfolio platform: Academics, Service, Leadership, and Scholarship. All of the identifying demographic data was blinded, and the octiles made it so that individual student contributions were not identified. This was all done to mitigate bias, so formal training was not done.

The selection criteria were defined across four domains. Leadership was characterized by holding positions in institutionally recognized student organizations. Scholarship was assessed through presentations and publications at local, regional, or national meetings, as well as peer-reviewed abstracts and manuscripts. Academic achievement was determined by class rank, with eligibility limited to students in the top 50% of the class and additional weight assigned to higher octiles. Service was quantified by the number of documented hours in service-related activities (e.g., civic organizations). Based on our review of internal data, these four categories capture the range of our students’ efforts and involvement. Moreover, these criteria were intentionally selected because our experience demonstrates there is a significant degree of participation across a variety of domains that help to equalize this effort.

Students’ grades from courses and clerkships in the first three years were used to generate a performance ranking, which was then divided into octiles. Only students in the top four octiles (i.e., the top half of the class) were included for further consideration. Scholarship octiles were assigned based on presentations and publications reported in MyPortfolio. Leadership and service were combined and used to produce a third octile. These three metrics (academics, 40%; scholarship, 20%; combined leadership and service, 40%) were weighted to generate a single value used to select the top 20% of the class for AΩA nomination.

Statistical analysis

Differences between AΩA members and nonmembers were compared using the χ2 test. Statistical analyses were performed using SPSS, Version 29.0.2.0 (IBM Corp., Armonk, USA). A 2-sided P < .05 defined statistical significance.

## Results

**Figure 1:**
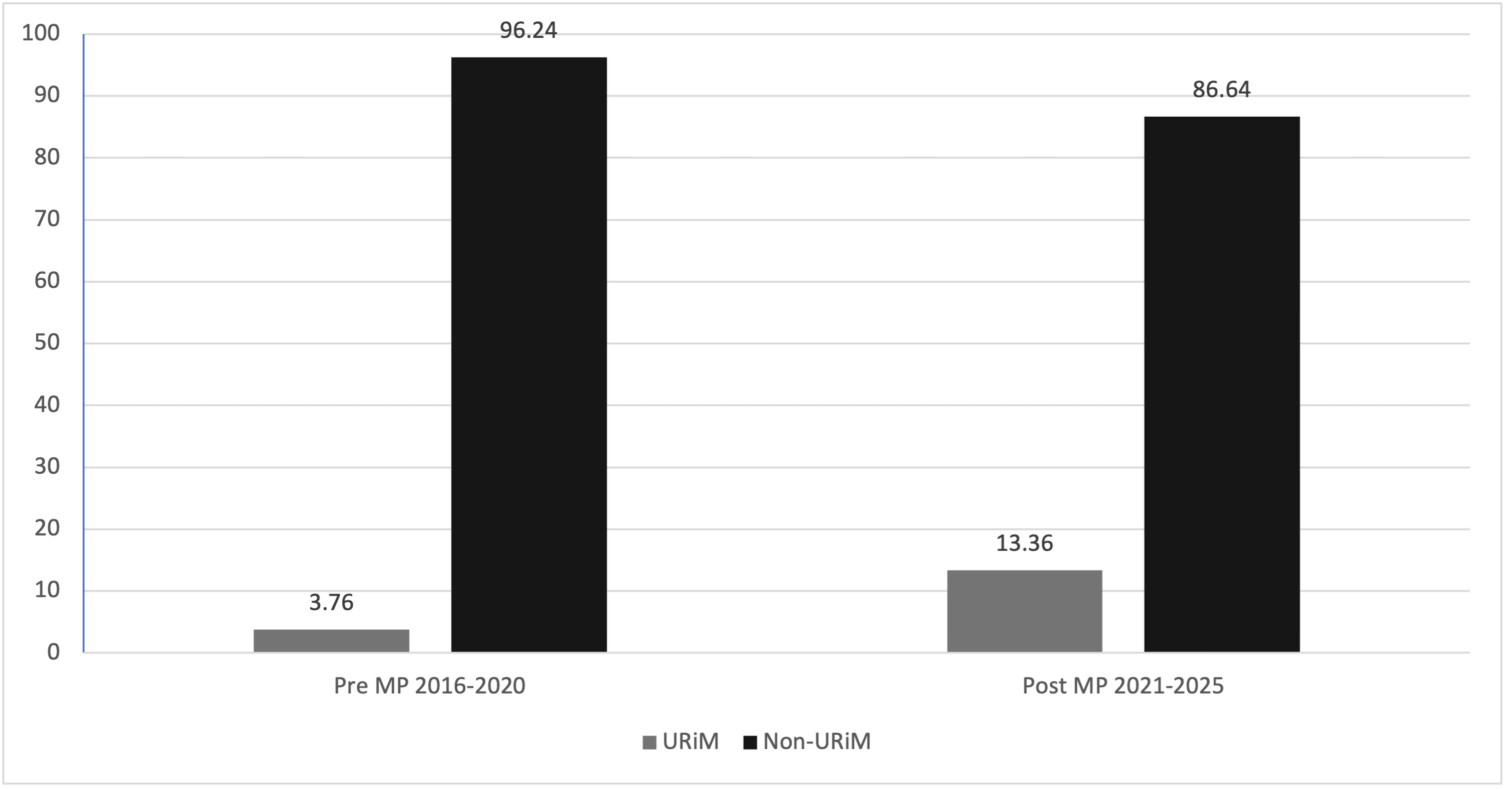
Percentage of Medical Student AΩA Electees Pre- and Post-MyPortfolio (MP) Implementation by Race and Ethnicity URiM: underrepresented in medicine

**Figure 2:**
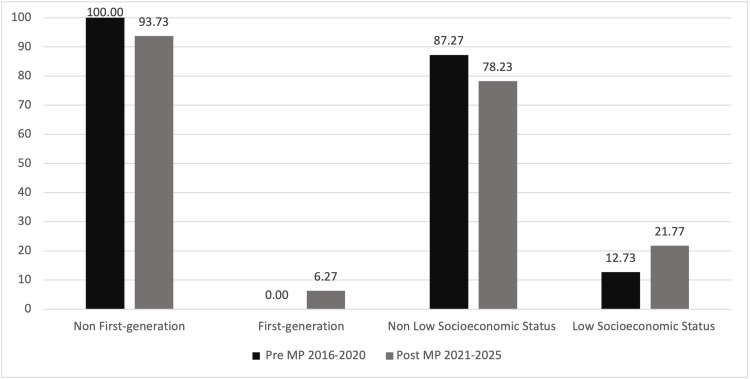
Percentage of Medical Student AΩA Electees Pre- and Post-MyPortfolio (MP) Implementation by First-Generation and Socioeconomic Status

Table 1 compares the demographic characteristics of 490 AΩA electees during the pre- (N = 220) and post-MP (N = 270) implementation periods. URiM students elected to AΩA increased from 3.76% to 13.36% from the pre- to post-MP period (χ²(1, N = 491) = 12.02, p < .01, v = 0.166) (Fig. 1). AΩA electees who identified as first-generation also increased from no representation (0%) in the pre-MP period to 6.27% in the post-MP period (χ² (1, N = 491) = 14.296 , p < .001, v = 0.171) (Fig. 2). There was an increase in students from lower SES backgrounds (SES1 and SES2) elected to AΩA (12.73% to 21.77%) from the pre- to post-MP period (χ² (1, N = 491) = 6.81, p < .001, v = 0.118) (Fig. 2). 

**Table 1:** Demographics of Medical Student AΩA membership Electees by Race and Ethnicity Pre- and Post-MyPortfolio (MP) Implementation

Race/Ethnicity	Pre-MP 2016–2020 % (n)	Post-MP 2021–2025 % (n)
American Indian or Alaska Native	0.45% (1)	1.11% (3)
Asian	20.45% (45)	27.41% (74)
Black or African American	0.45% (1)	4.81% (13)
White	68.18% (150)	52.22% (141)
Hispanic or Latino	1.82% (4)	6.67% (18)
More than one race or ethnicity	4.09% (9)	4.81% (13)
Did Not Answer	4.55% (10)	2.96% (8)
Total no. electees	220	270

## Discussion

Recent multi-institutional studies have documented persistent disparities in AΩA elections between students from non-URiM and URiM backgrounds, despite comparable academic performance [7-11]. These studies show the limitations of the traditional academic (GPA)-driven selection processes [11-14], and scholars have called for more nuanced, holistic approaches to reduce inequities [11] and broaden access to AΩA. Building on this work, our institution implemented a centralized, rubric-based review process within a standardized platform, My Portfolio (MP), to assess learner accomplishments, inclusive of academic achievement, depth of scholarly pursuits, and leadership and service to the community. To further mitigate bias, all individuals involved in the selection process were blinded reviewers. This model demonstrates a feasible alternative to the traditional academic approach. 

The findings indicate an increase in the election of students from historically disadvantaged backgrounds after a re-imagined holistic review of their achievements for AΩA election. With the changes to the AΩA charter [4], our institution used the opportunity to expand the selection criteria and emphasize leadership, service, scholarship, and academic excellence. The study’s main finding demonstrates that a more equitable and inclusive AΩA election can be achieved utilizing a centralized, standardized medical school platform (e.g., MP) to perform a holistic review. The results demonstrate an increase in student AΩA electees from URiM backgrounds [2], in addition to those from first-generation and lower SES backgrounds. First-generation students face specific challenges in medical school, including insufficient institutional support in navigating the hidden curriculum of medical education [15]. Students from lower SES backgrounds continue to face personal and educational-related challenges related to their familial income, which compounds the difficulties overcome in the admissions process [7].

Our results are therefore encouraging and highlight the importance of inclusive practices in medical education [8].

This study does have limitations. First, self-reported racial, ethnic, and socioeconomic status may not fully reflect the complexity of students’ backgrounds and experiences [9,10]. Second, our study involved a pre- and post-implementation analysis and may not completely reflect historical trends in AΩA membership. Another limitation is that this is a single-institution study, and our findings may not be replicable in other settings, particularly when considering possible reviewer subjectivity despite efforts (e.g., annual trainings) to address this issue. Recognizing that institutional culture and structure shape the student experience, the next phase of this work aims to expand its reach to include a multi-institution investigation. The goal would be to engage medical schools across the spectrum of size, type, and structure. The inclusion of public and private institutions will provide a more representative picture of the factors influencing student selection. 

Finally, while there were correlations between the new, holistic evaluation and representation of students, these findings cannot establish causality, as it is plausible that other institutional or demographic changes at our medical school could have acted as confounding variables, and may partly explain observed differences. The observed changes in student outcomes may reflect our expansion of engagement opportunities and emphasis on academic priorities through the evolution of the curriculum. Additionally, some of the patterns may simply reflect the random variability of year-to-year demographic shifts in the composition of the student body, which can impact the learning environment, academic performance, and participation patterns. However, the findings demonstrate an implementable process change aligned with AΩA’s charter that yields measurable shifts in recognition for students across multiple demographic domains (URiM, first-generation status, and SES). Furthermore, while it is clear that changes in demographics are supported by our holistic process selection, this study acknowledges that these changes can also be multifactorial in nature due to the dynamic interplay between evolving student composition and emerging academic priorities.

## Conclusions

Our findings demonstrate that implementing a centralized holistic review process for evaluating medical student achievement across leadership, scholarship, academic achievement, and service was effective in diversifying AΩA membership at our institution. By moving beyond grading metrics to a more holistic definition of academic excellence, we have successfully fostered a more inclusive recognition of talent and achievement. Moreover, the historically close linkage between AΩA membership and professional opportunities within medicine makes holistic review an important step towards diversifying the physician workforce and academic medicine. As we noted previously, the ability to access AΩA membership presents greater opportunities for career advancement. As such, it becomes imperative that holistic approaches are employed to align with the broader goals of academic medicine to cultivate a diverse, equitable, and dynamic physician workforce. Future research should explore the use of holistic evaluation methodologies in a multi-institutional and longitudinal context as further validity evidence.
